# Application of the New “Points in Range” Metrics in the Assessment of In-Hospital Glycemic Control

**DOI:** 10.7759/cureus.34278

**Published:** 2023-01-27

**Authors:** Patrícia Rosinha, Sofia Teixeira, Cláudia Amaral, Maria Helena Cardoso

**Affiliations:** 1 Department of Endocrinology, Centro Hospitalar do Baixo Vouga, Aveiro, PRT; 2 Department of Endocrinology, Centro Hospitalar Universitário do Porto, Porto, PRT

**Keywords:** points above range, points below range, points in range, capillary blood glucose monitoring, diabetes mellitus

## Abstract

Introduction

Capillary blood glucose (CBG) monitoring remains the most used testing form in hospitals and allows for “points in range (PIR)” metric calculation. This study was conceived to evaluate the metabolic control in patients with diabetes mellitus (DM) at a hospital through PIR metrics.

Methods

This was an observational cross-sectional study conducted on October 9, 2020, that included non-critical adults admitted to Centro Hospitalar Universitário do Porto (except pregnant/postpartum women) with DM under CBG monitoring and a minimum of 24 hours of hospitalization. Glycemic control was evaluated by previous day CBG monitoring.

Results

The study sample consisted of 110 patients with DM (93.6% type 2) with a median number of CBG tests of 4.00 (1.00) and a median CBG of 166.20 (69.41) mg/dL, SD 41.93 ± 27.20 mg/dL, and variation coefficient of 22.56 ± 12.51%. Points below range were 0.5%, with 0% below 54 mg/dL. The points in ranges 70-140 mg/dL and 140-180 mg/dL were 32.8% and 22.0%, respectively, and the total number of patients with all points in range 70-180 mg/dL was 19 (17.3%), with only 3 (2.7%) having all points in range 140-180 mg/dL and 10 (9.1%) in range 70-140 mg/dL. Regarding points above range (PAR), 29.9% and 14.8% points were at levels 1 and 2 hyperglycemia, respectively, and 15 (13.6%) patients had all points above 180 mg/dL. Correlations were identified between PAR and the total number of CBG assessments (ρ = 0.689, p < 0.001).

Conclusion

We conclude that in-hospital glycemic control remains suboptimal: only few have adequate control according to the PIR metrics despite low glycemic variability. PIR metrics are a new, valuable, simple and valid way to take better advantage of CBG monitoring at no added cost.

## Introduction

Capillary blood glucose (CBG) and continuous glucose monitoring (CGM) provide different information about the glucose regulatory situation of a certain patient. CBG monitoring provides an accurate static value that reflects glucose concentration in the body’s transport system but does not report on its variations over time. Instead, CGM offers a dynamic measurement of the interstitial fluid glucose levels. These two forms measure glucose levels in different compartments, which can be associated with some degree of discrepancy especially in situations of high glycemic variability [1].

We have been witnessing the exponential growth in forms of CGM worldwide and this has led to the emergence of the concepts of glycemic variability (GV), time in range (TIR), time above range (TAR), time below range (TBR) and other CGM metrics that complement the value of HbA1c [2,3]. In 2019, the Advanced Technologies and Treatments for Diabetes (ATTD) Congress panel published guidance on CGM-based targets for the assessment of glycemic control for different diabetic populations using those metrics [4].

Nonetheless, CBG testing remains the most widely used form of inpatient monitoring because it is associated with lower costs and there are some doubts about CGM accuracy in hospitalized patients. However, despite the usefulness of CBG in daily decision making in hospitalized patients, the evaluation of the global metabolic control in these patients is difficult to assess based on individual CBG testing. During the COVID-19 pandemic, there was a need to develop strategies for the remote monitoring of glucose in hospitalized patients, which led to greater liberalization of the in-hospital temporary use of CGM. After April 2020, when the Food and Drug Administration (FDA) made the use of CGM available in hospitalized COVID-19 patients, several studies sought to assess its accuracy and safety in inpatient settings with favorable results, although there is still not enough evidence to prove the cost-benefit of its more indiscriminate use in this context [5]. Therefore, it is urgent to find alternative ways of obtaining the complementary information provided by CGM in situations where its widespread use is not possible.

The analysis of self-monitoring CBG values by Diabetes Management Systems (DMS) led to the perception that those isolated values could be pooled to provide additional information on glycemic control, similarly to what is done with CGM systems. This way, these new CBG monitoring metrics, the “points in range (PIR)”, were created seeking to bypass its limitations compared to CGM but considering that CBG only provides isolated glucose values. The PIR metrics are interpreted similarly to CGM’s TIR and provide additional information on the isolated value of CBG [6].

The primary objective of this study was to describe CBG monitoring in PIR metrics and to assess the metabolic control in patients with diabetes mellitus (DM) in the hospital through PIR metrics. Furthermore, we also intended to compare PIR metrics in the infection/non-infection subgroups and with respect to the length of hospitalization.

The results of this study were previously presented as a meeting abstract at the Portuguese Congress of Endocrinology (72nd Annual Meeting of the Portuguese Society of Endocrinology, Diabetes and Metabolism), January 29-31, 2021.

## Materials and methods

This was an observational cross-sectional study conducted on October 9, 2020, at Centro Hospitalar Universitário do Porto, a central hospital in Portugal, and included non-critical adult patients (except pregnant/postpartum women) with diagnosis of DM (by consulting the clinical process), hospitalized for a minimum of 24 hours and under CBG monitoring. A total of 31 patients with insufficient clinical information in the process were excluded. The study protocol conformed to the World Medical Association’s Helsinki Declaration and was approved by the Ethics Committee of Centro Hospitalar Universitário do Porto, approval number 2021.079 (065-DEFI/068-CE). Informed consent was waived by the Ethics Committee based on the retrospective nature of the study and full data anonymization. The following data were collected from the electronic clinical record: demographic information, day of hospitalization, main diagnosis (infection/non-infection), type of DM and its microvascular/macrovascular complications, and CBG monitoring of the previous day.

To define the total points in each of the intervals, we took into account both the American Diabetes Association (ADA) recommendations regarding glycemic control in hospitalized patients and the CGM-based targets defined in the 2019 ATTD consensus [4,7]. This way, we considered points below range (PBR) as <70 mg/dL, PIR as 70-180 mg/dL and points above range (PAR) as >180 mg/dL, calculated by adding the number of CBG tests in each of the periods. For example, a patient who had the CBG test results 181, 230, 74, 140 mg/dL would have a total of 0 PBR, 2 PIR and 2 PAR. Similarly, the percentage of points per interval for each patient was calculated by dividing the number of CBG tests in each range by its total amount in that 24-hour period and multiplying it by 100. In the previous example, we would have the following percentages: 0% PBR, 50% PIR and 50% PAR.

GV was assessed by standard deviation (SD) of the mean glucose value of each patient and the value of coefficient of variation (CV) (calculated by the formula: SD/mean glucose x 100) [3]. An analysis of subgroups was carried out in order to compare PIR metrics in the infection/non-infection groups, and in accordance with the length of hospitalization (categorized as 1-3 days, 4-7 days, 8-14 days, 15-30 days and >30 days).

Data analysis was performed using the statistical package IBM SPSS Statistics, version 20.0.0 (IBM Corp., Armonk, NY). Categorical variables were presented as frequencies and percentages, and continuous variables as means and SD or medians and interquartile ranges (IQR) for variables with skewed distributions. Normal distribution was checked using the Shapiro-Wilk test or skewness and kurtosis as appropriate. All reported p-values are two-tailed, with a p < 0.05 indicating statistical significance. Spearman’s correlation coefficient (ρ) was used to assess the correlation between PIR metrics and other parameters under analysis, namely, the length of hospital stay and the number of CBG tests. The interpretation of correlations’ strength was made using the reference values ​​provided by Bryman and Cramer [8]. For the analysis by main diagnosis and day of hospitalization subgroups, we used Mann-Whitney U-test and Kruskal-Wallis test, respectively.

## Results

The sample description is shown in Table 1, consisting of 110 patients with a predominance of males (60.0%), an average age of 72.70 ± 11.49 years and a median hospital stay of 11.00 (17.00) days at the time of data collection. The most frequent type of DM was type 2 in 93.6%, and with regard to complications, 67 (60.9%) patients had at least one documented microvascular or macrovascular complication and 41.8% had a diagnosis of infection (Table 1). The median number of CBG tests of the previous day was 4.00 (1.00).

**Table 1:** Sample description DM, diabetes mellitus; GC, glucocorticoids; IQR, interquartile range; SD, standard deviation

Sample characteristics	n (%)	
Gender		
Male	66	(60.0)
Female	44	(40.0)
Age at time of data collection (years), mean ± SD	72.70 ± 11.49	
Minimum-maximum	32-92	
Median hospital stay (days), median (IQR)	11.00 (17.00)	
Day of hospitalization		
1-3 days	22	(17.2)
4-7 days	22	(17.2)
8-14 days	32	(25.0)
15-30 days	30	(23.4)
>30 days	22	(17.2)
DM classification		
Type 1	4	(3.6)
Type 2	103	(93.6)
Induced by GC	2	(1.8)
Not yet clarified	1	(0.9)
Microvascular complications		
0	68	(61.8)
1	28	(25.5)
2	10	(9.1)
3	4	(3.6)
Macrovascular complications		
0	62	(56.4)
1	36	(32.7)
2	10	(9.1)
3	2	(1.8)
Main diagnosis		
Infection	46	(41.8)
Non-infection	64	(58.2)

Table 2 summarizes glycemic control results, including those of the new PIR metric application in the study population. The median blood glucose value was 166.20 (69.41) mg/dL and the mean SD and CV were 41.93 ± 27.20 mg/dL and 22.56 ± 12.51%, respectively. PBR were 0.5%, with 0% below 54 mg/dL. Points in the intervals 70-139 mg/dL and 140-180 mg/dL were 32.8% and 22.0%, respectively. Regarding PAR, 29.9% and 14.8% were at levels 1 and 2 of hyperglycemia, respectively (Table 2). The total number of patients with all points in the range 70-180 mg/dL was 19 (17.3%), with only 3 (2.7%) having all points in the range 140-180 mg/dL and 10 (9.1%) in the range 70-139 mg/dL. In what concerns PAR, the total of patients with all points above 180 mg/dL was 15 (13.6%).

**Table 2:** Glycemic control description CBG, capillary blood glucose; HbA1c, glycated hemoglobin; IQR, interquartile range; SD, standard deviation

Results		
Number of CBG tests, n (%)		
2	4	(3.6)
3	33	(30.0)
4	58	(52.7)
5	11	(10.0)
6	4	(3.6)
Minimum CBG value (mg/dL), median (IQR)	124.00	(45.00)
Maximum CBG value (mg/dL), mean ± SD	226.90 ± 77.25	
HbA1c (%), median (IQR)	7.20	(2.20)
Points per glucose range, n (%)		
<54 mg/dL	0	
54-69 mg/dL	2	(0.5)
70-139 mg/dL	137	(32.8)
140-180 mg/dL	92	(22.0)
181-250 mg/dL	125	(29.9)
>250 mg/dL	62	(14.8)
Total	418	(100.0)

Table 3 shows the results of the correlation analysis. A positive, statistically significant and moderate correlation was identified between the total of PAR and the number of CBG assessments (Table 3).

**Table 3:** Correlation analysis CBG, capillary blood glucose; HbA1c, glycated hemoglobin; PAR, points above range; PIR, points in range; ρ, Spearman's correlation

	PIR		PAR	
	(70-180 mg/dL)		(>180 mg/dL)	
	Correlation coefficient (ρ)	p value	Correlation coefficient (ρ)	p value
Number of CBG tests	-0.094	0.549	0.684	<0.001
Days of hospitalization	0.222	0.152	-0.139	0.508

Regarding the subgroup analysis by main diagnosis (infection/non-infection), we found a greater number of CBG tests in the infection group, 4.0 (0.0) vs. 3.0 (1.0), p = 0.011, although there were no significant differences in relation to the total PIR, 3.0 (2.0) vs. 3.0 (1.0), p = 0.910, or PAR, 3.5 (2.0) vs. 3.0 (1.0), p = 0.680. With respect to the length of stay subgroup analysis, no differences were found in terms of the number of CBG tests (p = 0.062), total PIR (p = 0.167) or PAR (p = 0.199).

## Discussion

Using the 2019 ATTD consensus, these results showed that inpatient glycemic control remained suboptimal because only a minority of the patients had adequate control according to PIR metrics: the total of points in the range 70-180 mg/dL was 54.8% with a minimum percentage of PBR, which means that hyperglycemia continues to keep us from reaching our glycemic targets. This is supported by the low percentage of patients with all points in the range 70-180 mg/dL, thus indicating that the number of patients with at least one assessment corresponding to hyperglycemia is quite high.

We found a positive significant and moderate correlation between CBG tests and PAR. One possible explanation might be the tendency to intensify CBG monitoring in face of poor glycemic control. Yet, the opposite would be expected for PIR, that is, a negative correlation between the number of CBG tests and the total of PIR, which was not verified. Thereby, the results of the subanalysis by main diagnosis are also interpretable along this line of thinking: there was a significantly higher number of CBG tests in the infected group in which worse glycemic control would be expected, but probably not sufficiently higher to reflect a difference in the total PIR/PAR. Besides, the lack of correlation between PIR/PAR and length of stay combined with the absence of significant differences in the subanalysis by day of hospitalization suggests that the length of stay might be a less important factor compared to infection diagnosis to take into account as a potential interfering factor in glycemic control.

This was the first study to apply the PIR metrics to inpatients and demonstrate its relevance in a real context for the assessment of glycemic control. Despite the existence of FDA-approved CGM devices for non-critical patients, their widespread use is not possible in clinical practice mainly for reasons of cost [9]. It was proved by this study that the calculation of PIR metrics is a possible, simple and inexpensive way to obtain information complementary to the isolated glucose values obtained by CBG monitoring at no added cost. Nevertheless, in order to take better advantage of this metric, it would be desirable to have correlations between the PIR and CGM metrics and also recommendations regarding the appropriate intervals and the respective percentages of points in each of the intervals, similar to ATTD consensus recommendations [4]. Moreover, given the exponential growth in the number of outpatient CGM users, it would be desirable that these recommendations exist in particular for the inpatient situation.

As limitations of this study, the following should be noted: it was cross-sectional in nature that only allowed a punctual assessment of glycemic control by this new metrics; the sample size may still have been insufficient to obtain significant correlations between some variables under study and the PIR metrics. In addition, it is also worth to mention that this study was carried out in hospitalized patients and the use of CBG monitoring in this population has limitations, because numerous factors, such as the patient's perfusion status, can influence the accuracy of this measurement [10].

## Conclusions

In light of the results found in this study, in-hospital glycemic control seems to remain suboptimal: only few have adequate control despite low glycemic variability, which points to the relevance of finding ways to improve it without additional costs. In conclusion, PIR metrics are a new, valuable, simple and valid way to take better advantage of CBG monitoring and overcoming its limitations at no added cost. Further longitudinal studies are still desirable to confirm and eventually extend these results.
